# Supplementary material for: Apo and Aβ46-bound γ-secretase structures provide insights into amyloid-β processing by the APH-1B isoform
Source: Nat Commun. 2024 May 27;15:4479. doi: 10.1038/s41467-024-48776-2 (PMC11130327; doi:10.1038/s41467-024-48776-2)
Supplement: Supplementary file 1 — Supplementary Information [file 41467_2024_48776_MOESM1_ESM.pdf]

SUPPLEMENTARY INFORMATION

Apo and Ab46-bound γ-secretase structures provide insights into amyloid-β processing by the APH-1B isoform

Ivica Odorcic[1,2,3,4], Mohamed Belal Hamed[3,4], Sam Lismont[3,4], Lucia Chavez Gutierrez[3,4*$], and Rouslan G. Efremov[1,2*$]

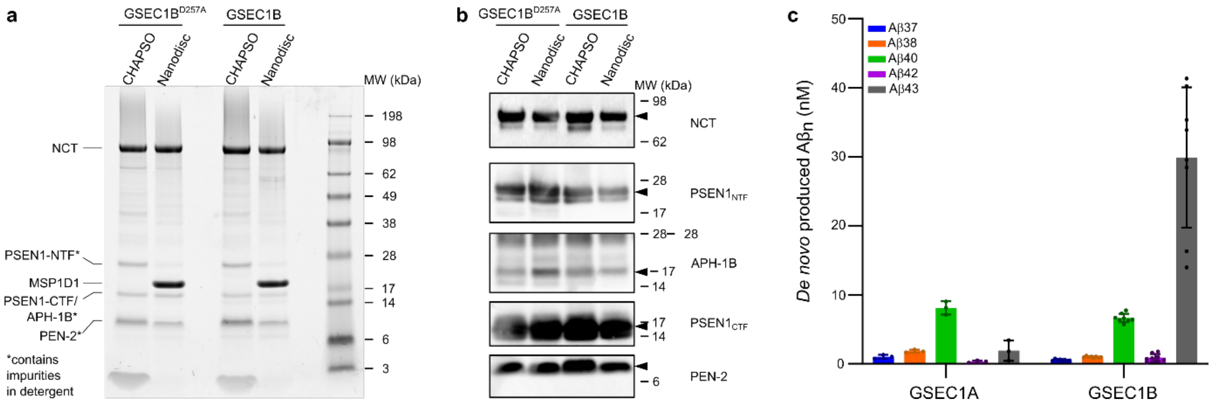

Supplementary Figure 1. Characterization of GSEC1 reconstituted into lipid nanodiscs. a, SDS-PAGE of GSEC1B and GSEC1B^{D257A} purified in CHAPSO and reconstituted into nanodiscs. **b,** Western blot of the same samples as in a. **c,** The amount of Aβ measured from activity assays done with GSEC1A and GSEC1B reconstituted into nanodiscs. Data are presented as mean ± SD, n=3 for GSEC1A and n=8 for GSEC1B. Source data for panel a and b are provided at the end of the Supplementary Information. Source data for panel c are provided as a Source Data file.

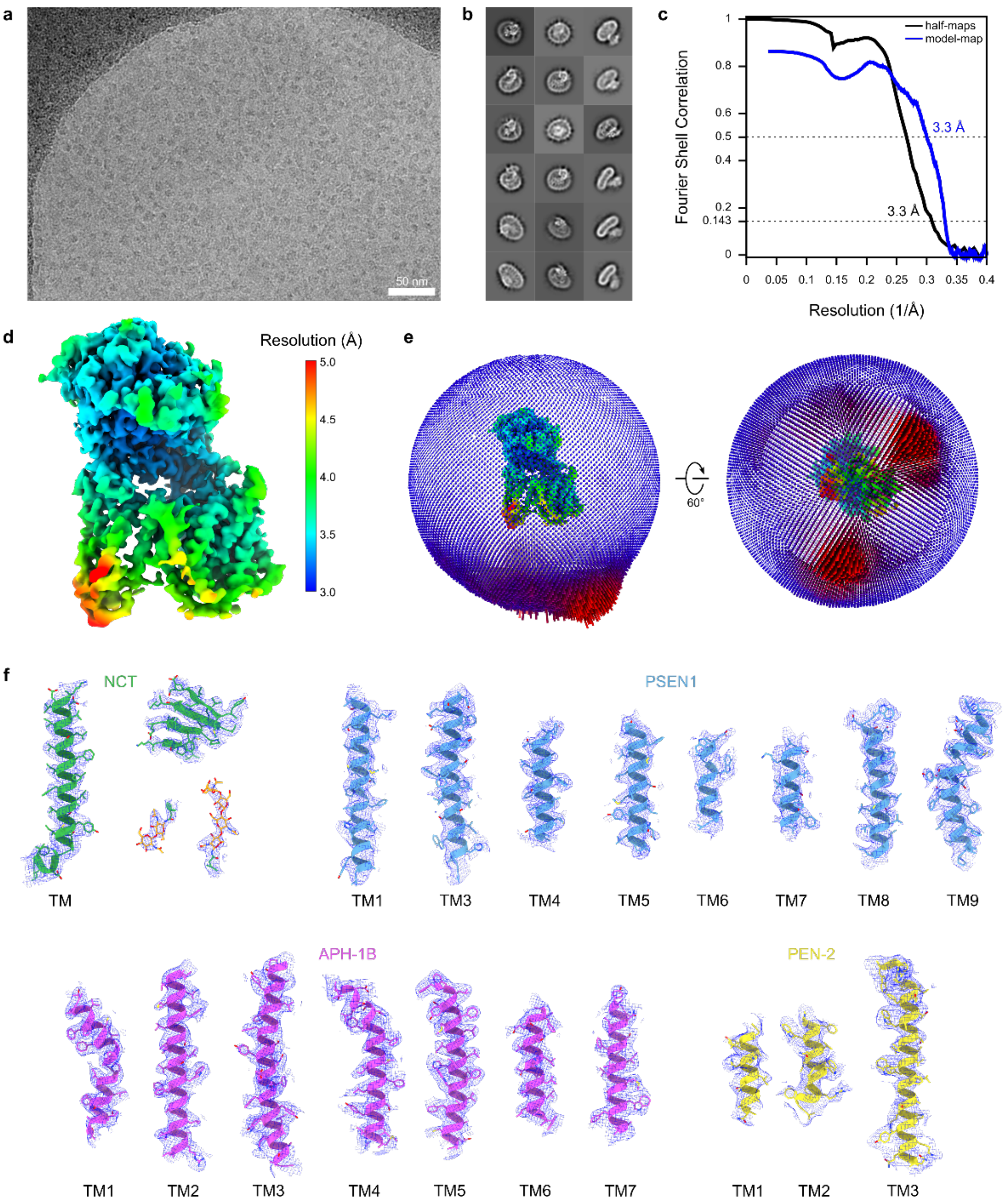

Supplementary Figure 2. Electron microscopy and single-particle analysis of apo GSEC1B. a, Representative micrograph from the apo GSEC1B dataset after motion correction, n > 1000. **b,** Example 2D class averages calculated in RELION 3.1. **c,** Masked half-map and model-map Fourier shell correlation curves. The resolution cut-offs at FSC of 0.143 and 0.5 are indicated for half-map and model-map FSCs. **d,** EM density map coloured by local resolution. **e,** Distribution of particle orientations. **f**, Examples of EM density maps (shown as mesh surfaces) around the atomic models (shown as cartoons and sticks).

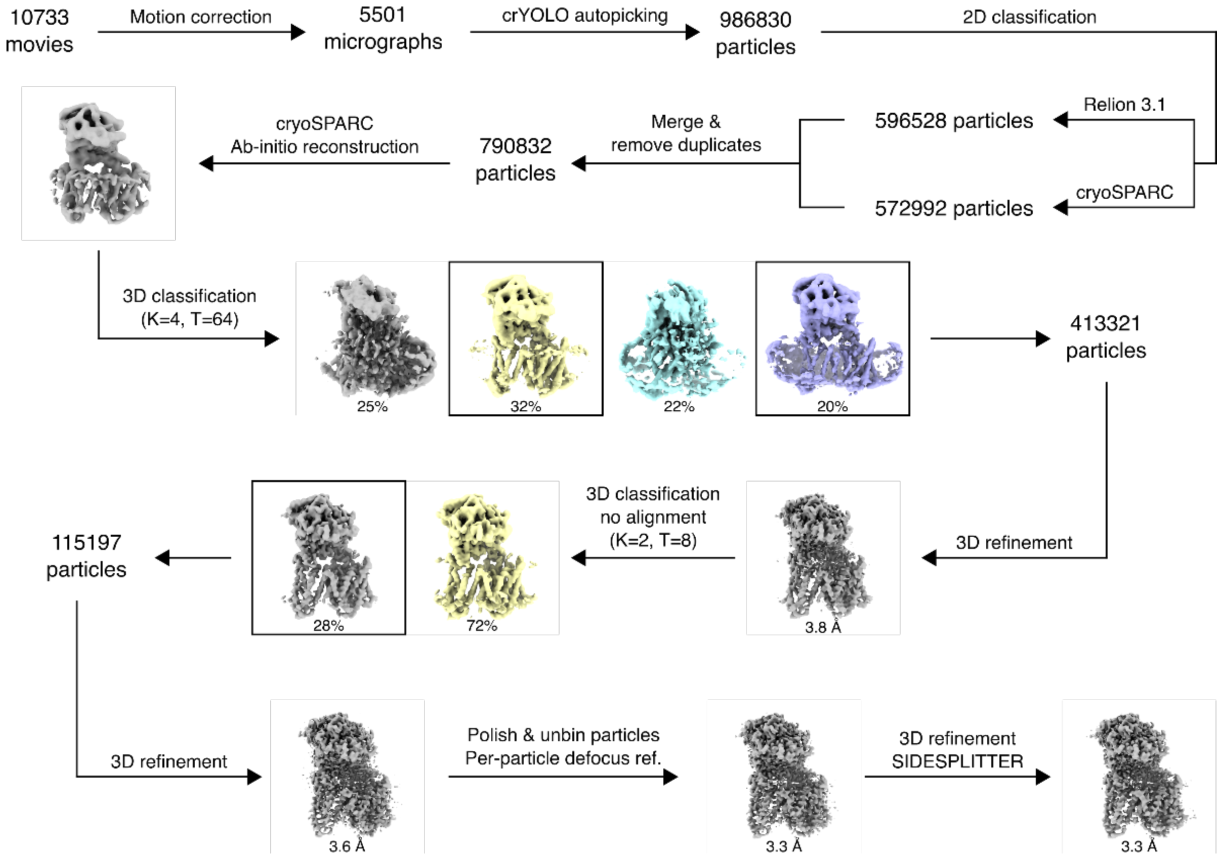

Supplementary Figure 3. Image processing pipeline for apo GSEC1B. Schematic representation of cryo-EM data processing procedure used for obtaining reconstruction of GSCE1B in apo form.

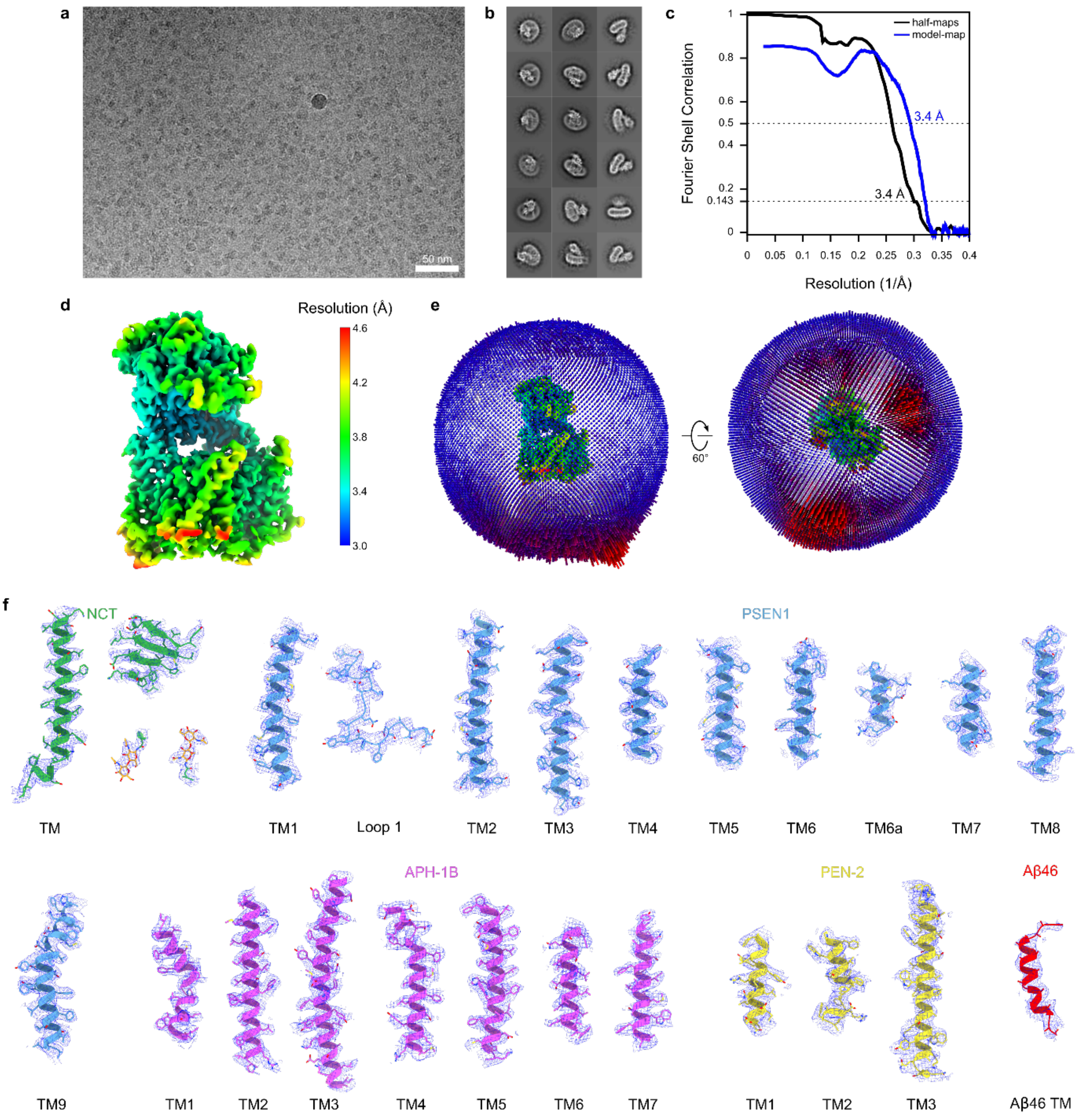

Supplementary Figure 4. Electron microscopy and single-particle analysis of GSEC1B-Aβ46 complex. a, Representative motion-corrected micrograph from the GSEC1B-Aβ46 dataset, n > 1000. **b,** Example 2D class averages calculated in RELION 3.1. **c,** Fourier shell correlation curves for masked half-maps and between model and map. The resolution cut-offs at FSC of 0.143 and 0.5 are indicated for half-map and model-map FSCs. **d,** EM density map coloured by local resolution. **e,** Distribution of particle orientations. **f,** Examples of EM density maps (shown as mesh surfaces) around atomic models (shown as cartoons and sticks).

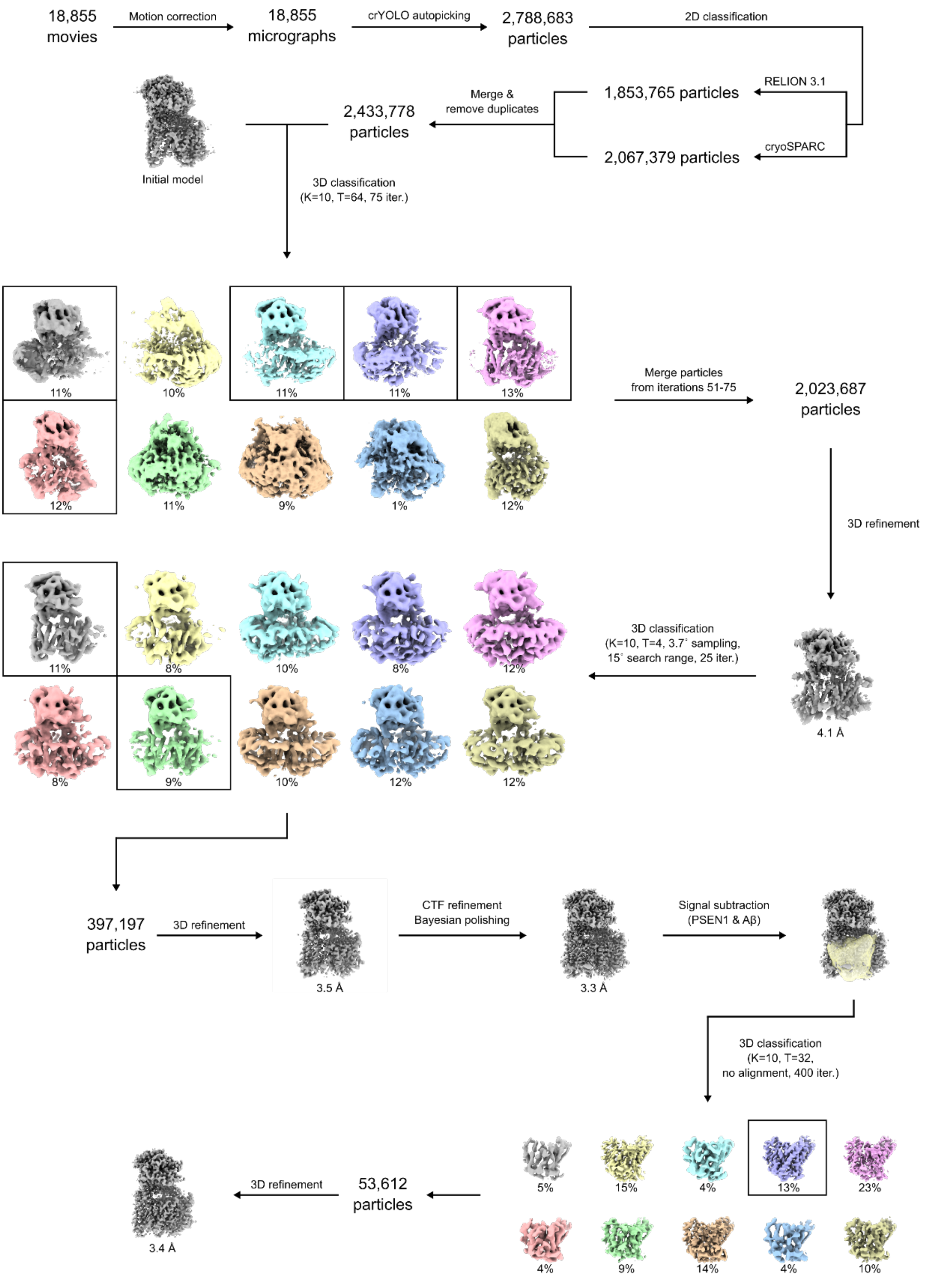

Supplementary Figure 5. Image processing pipeline for GSEC1B-Aβ46 complex. Schematic representation of cryo-EM data processing procedure used for obtaining reconstruction of GSCE1B-Aβ46.

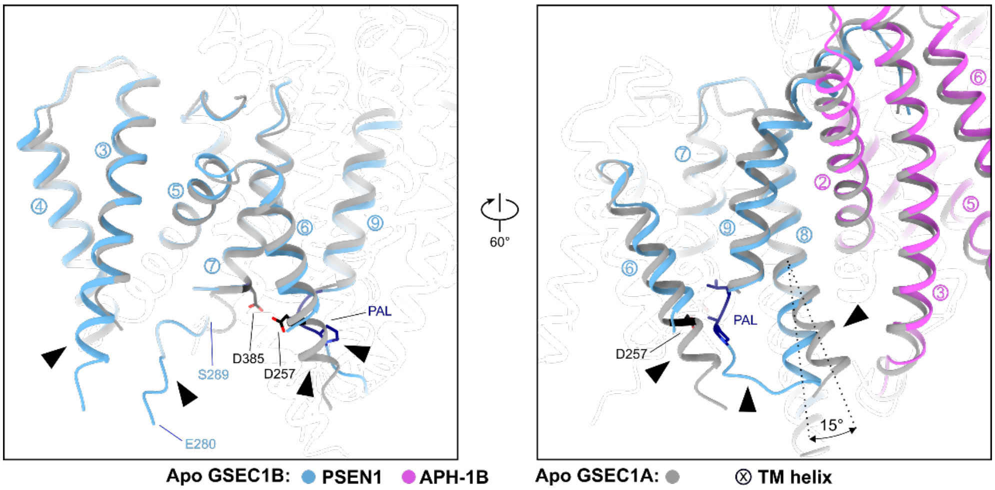

Supplementary Figure 6. Conformational differences in PSEN1 between GSEC1A and GSEC1B in apo states. Structural alignment of GSEC1B and GSEC1A (PDB: 5FN5). PSEN1 and APH-1 are shown as cartoons; the catalytic residues and the PAL motif are shown as sticks. The arrows indicate the main conformational differences.

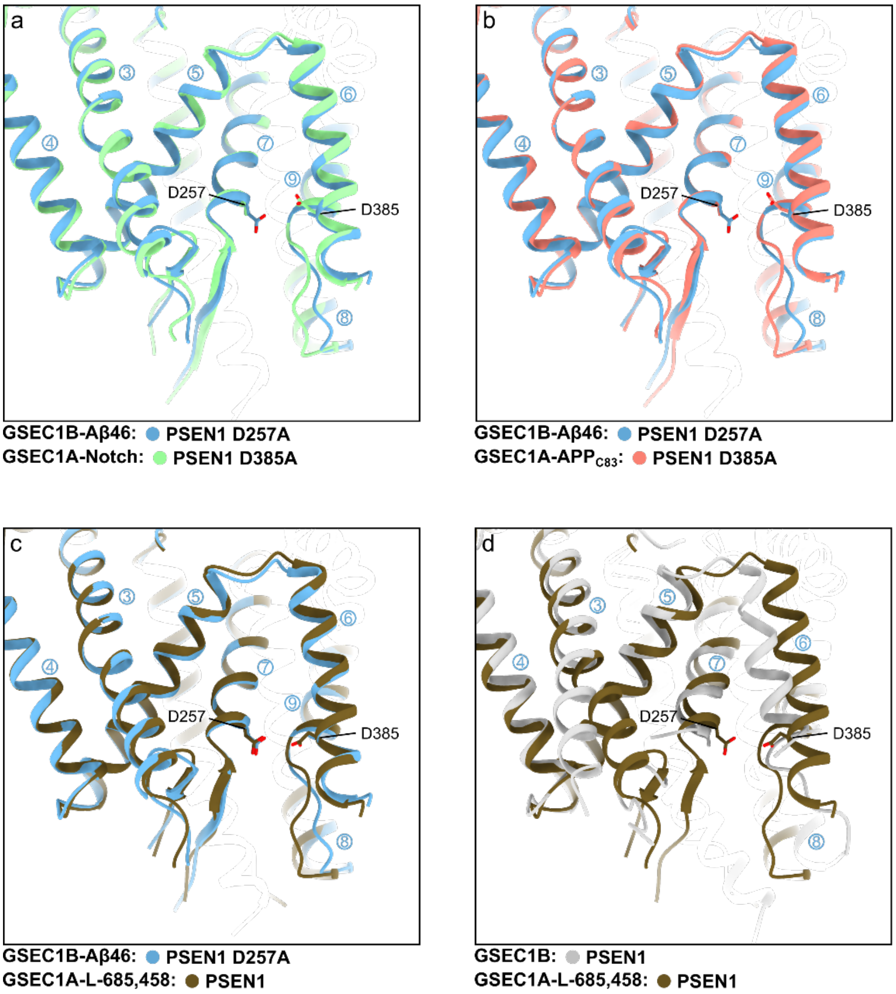

Supplementary Figure 7. Comparison of active sites of WT and active-site-mutated PSEN1 structures. Structural alignment of: **a)** $PSEN1^{D257A}$ from GSEC1B in complex with Aβ46 (this paper) and $PSEN1^{D385A}$ from GSEC1A in complex with Notch (PDB: 6IDF); **b)** $PSEN1^{D257A}$ from GSEC1B in complex with Aβ46 (this paper) and $PSEN1^{D385A}$ from GSEC1A in complex with APP_{C83} (PDB: 6IYC); **c)** $PSEN1^{D257A}$ from GSEC1B in complex with Aβ46 (this paper) and PSEN1 from GSEC1A in complex with the transition state analogue inhibitor L-685,458 (PDB:7C9I); **d)** PSEN1 from GSEC1B in apo form (this paper) and PSEN1 from GSEC1A in complex with the transition state analogue inhibitor L-685,458 (PDB:7C9I). The structures are shown as cartoons with the catalytic residues shown as sticks. PSEN1 TM helices are indicated with circled numbers.

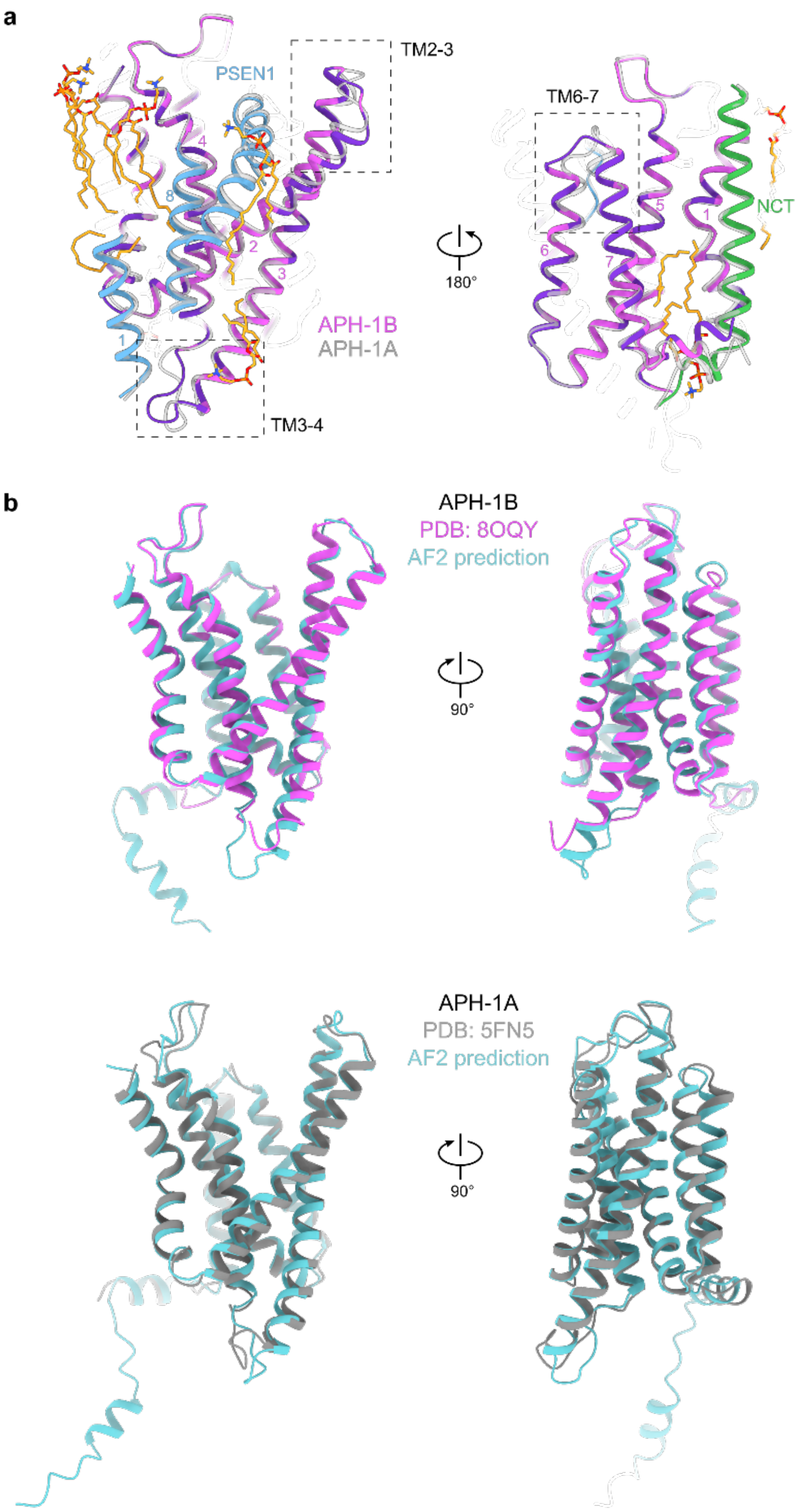

Supplementary Figure 8. Comparison of APH-1 isoforms in substrate-bound structures with APH-1 structures predicted by AlphaFold2. a, Structural differences between APH-1 isoforms from the GSEC1B-Aβ46 and GSEC1A-APP$_{C83}$ structures. Non-conserved APH-1B TM domain residues at the interface with PSEN1 are shown in purple. **b,** APH-1A and APH-1B structures as predicted by AF2, retrieved from UniProt (entries Q96BI3-F1-model_v4 and Q8WW43-F1-model_v4).

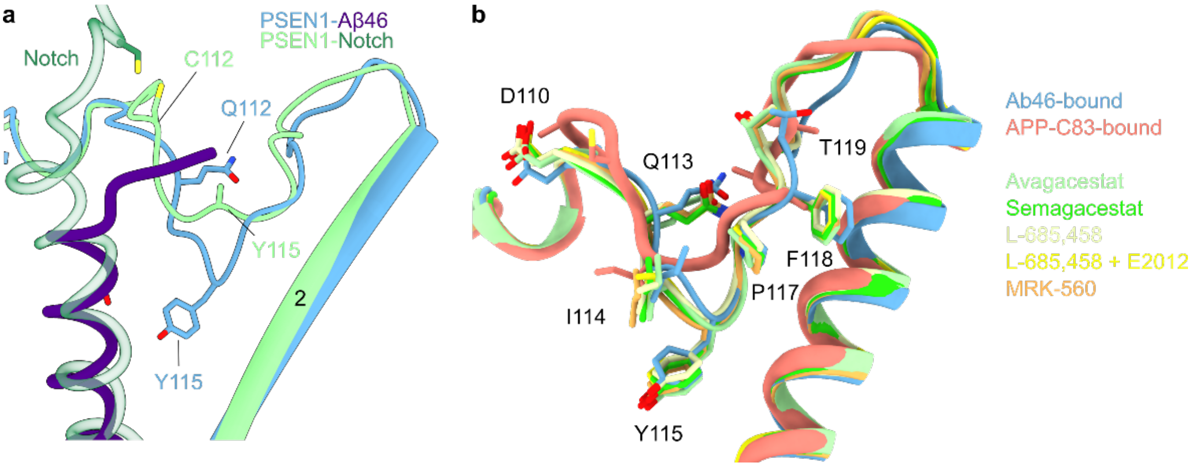

Supplementary Figure 9. Comparison of loop1^{PSEN1} conformations between GSEC structures. a, GSEC1A-Notch (PDB:6IDF) structure is shown aligned to GSEC1B-Aβ46 structure. Side chains mutated to cysteines to form disulphide crosslinking (P9C on Notch and Q112C on PSEN1) are shown as sticks. **b,** Alignment of loop 1 from several GSEC structures solved in the presence of inhibitors[73–75]. The inhibitors used are indicated on the right and colour coded to match the structures. Loop 1 backbone is shown as cartoon, and selected residues are shown as sticks.

Supplementary Table 1. Sequence modelled for individual GSEC subunits. The model completeness is shown for GSEC1A, columns 1 and 3, and GSEC1B solved in this work, columns 2 and 4.

	**GSEC1A apo (PDB: 5FN5)**	**GSEC1B apo (PDB: 8OQY)**	**GSEC1B-APP$_{C83}$ (PDB:6IYC)**	**GSEC1B-Aβ46 (PDB: 8OQZ)**
NCT	34-698 (94%)	34-699 (94%)	34-700 (94%)	34-610, 613-700 (94%)
PSEN1	68-108, 167-264, 379-429, 435-467 (48%)	73-107, 160-256, 280-289, 383-467 (49%)	73-291, 376-467 (67%)	73-291, 376-467 (67%)
APH-1A	2-244 (92%)	-	2-244 (92%)	-
APH-1B	-	2-104, 111-238 (90%)	-	2-241 (93%)
PEN-2	2-21, 25-44, 46-101 (95%)	2-101 (99%)	2-101 (99%)	2-101 (99%)

Supplementary Table 2. RMSD between aligned structures of GSEC1A and GSEC1B isoforms.

GSEC1B PDB: 8OQY	GSEC1A PDB: 5FN5	RMSD (Å)	Nr. of atoms
Overall	Overall	1.2	7918
Nicastrin	Nicastrin	0.9	4416
PSEN1	PSEN1	1.3	1353
APH-1B	APH-1A	0.9	1283
PEN-2	PEN-2	1.1	646

GSEC1B PDB: 8OQY	GSEC1B-Aβ46 PDB: 8OYZ	RMSD (Å)	
Overall	Overall	0.8	7365
Nicastrin	Nicastrin	0.4	4127
PSEN1	PSEN1	1.5	1316
APH-1B	APH-1B	0.4	1332
PEN-2	PEN-2	0.6	580

GSEC1B-Aβ46 PDB: 8OQZ	GSEC1A-APP$_{C83}$ PDB: 6IYC	RMSD (Å)	
Overall	Overall	0.8	8449
Nicastrin	Nicastrin	0.5	4264
PSEN1	PSEN1	0.6	1823
APH-1B	APH-1A	0.7	1340
PEN-2	PEN-2	0.6	654
**Aβ46**	APP-C83	1.0	85

GSEC1A PDB: 5FN5	GSEC1A-APP$_{C83}$ PDB: 6IYC	RMSD (Å)	
Overall	Overall	1.3	7808
Nicastrin	Nicastrin	0.8	4457
PSEN1	PSEN1	2.1	1431
APH-1A	APH-1A	0.9	1530
PEN-2	PEN-2	1.2	694

Supplementary Table 3. List of APH-1 residues located at the interface with PSEN1 subunit.

APH-1A	APH-1B	Change APH-1A -> APH-1B	PSEN1 sidechains within 4.5 Å	
			APH-1A	APH-1B
V32	I32	Longer	F86	F86
V36	I36	Longer	F86, L415, I416, C419, L420	F86, V412, L415, I416
V51	L51	Longer	L452	L452
R62	N62	Positive -> polar	Q459	-
Y69	K69	Aromatic -> positive	-	F465
L104	I104	-	E72	A79
D107	G107	Positive -> no sidechain	L73, K76	-
I128	M127	Longer	V412	V412
I135	V134	Shorter	W404, N405	W404
I137	T136	Hydrophobic -> polar	A461	A461
Y156	F155	Polar -> hydrophobic	Q464	Q464
T159	Y158	Liner -> aromatic	Q464	Q464, Y466
L163	M162	Longer	Y466	Y466
T200	V199	Polar -> hydrophobic	-	-
N207	S206	Shorter	Q464, F465, Y466	-

Supplementary Table 4. Cα-Cα distances between catalytic residues

GSEC	PDB ID	Substrate	D257-D385 Cα-Cα distance (Å)	Notes
**GSEC1B**	8OQY	-	-	D257 is not modelled
**GSEC1B D257A**	8OQZ	Aβ46	10.0	
**GSEC1A**	5FN5	-	9.1	
**GSEC1A D385A**	6IDF	Notch	10.4	
**GSEC1A D385A**	6IYC	APP_{C83}	10.6	
**GSEC**	**PDB ID**	**Inhibitor**	**D257-D385 Cα-Cα distance (Å)**	**Notes**
**GSEC1A**	5FN2	DAPT	8.0	
**GSEC1A**	6LQG	Avagacestat	10.5	
**GSEC1A**	6LR4	Semagacestat	10.0	
**GSEC1A**	7C9I	L-685,458	10.2	
**GSEC1A**	7Y5T	MRK-560	10.5	
