# Supplementary material for: Apo and Aβ46-bound γ-secretase structures provide insights into amyloid-β processing by the APH-1B isoform
Source: Nat Commun. 2024 May 27;15:4479. doi: 10.1038/s41467-024-48776-2 (PMC11130327; doi:10.1038/s41467-024-48776-2)
Supplement: Supplementary file 3 — Description of Additional Supplementary Files [file 41467_2024_48776_MOESM3_ESM.docx]

**Description of Additional Supplementary Files**

**Supplementary Movie 1:** Conformational changes between GSEC1B in nanodiscs and GSEC1A in amphipols.

**Supplementary Movie 2:** Conformational changes between GSEC1B in the apo and Aβ46-bound state.

**Supplementary Movie 3:** Conformational changes between GSEC1B-Aβ46 and GSEC1A-APPC83.

**Supplementary Data 1:** List of primer sequences used to clone PSEN1 mutants.
