# Supplementary material for: Apo and Aβ46-bound γ-secretase structures provide insights into amyloid-β processing by the APH-1B isoform
Source: Nat Commun. 2024 May 27;15:4479. doi: 10.1038/s41467-024-48776-2 (PMC11130327; doi:10.1038/s41467-024-48776-2)
Supplement: Supplementary file 8 — Reporting Summary [file 41467_2024_48776_MOESM8_ESM.pdf]

nature portfolio

Corresponding author(s): Rouslan Efremov and Lucia Chavez-Gutierrez

Last updated by author(s): Apr 16, 2024

Reporting Summary

Nature Portfolio wishes to improve the reproducibility of the work that we publish. This form provides structure for consistency and transparency in reporting. For further information on Nature Portfolio policies, see our Editorial Policies and the Editorial Policy Checklist.

Statistics

For all statistical analyses, confirm that the following items are present in the figure legend, table legend, main text, or Methods section.

n/a	Confirmed	
☐	☒	The exact sample size (*n*) for each experimental group/condition, given as a discrete number and unit of measurement
☐	☒	A statement on whether measurements were taken from distinct samples or whether the same sample was measured repeatedly
☐	☒	The statistical test(s) used AND whether they are one- or two-sided<br>*Only common tests should be described solely by name; describe more complex techniques in the Methods section.*
☒	☐	A description of all covariates tested
☒	☐	A description of any assumptions or corrections, such as tests of normality and adjustment for multiple comparisons
☐	☒	A full description of the statistical parameters including central tendency (e.g. means) or other basic estimates (e.g. regression coefficient) AND variation (e.g. standard deviation) or associated estimates of uncertainty (e.g. confidence intervals)
☐	☒	For null hypothesis testing, the test statistic (e.g. *F*, *t*, *r*) with confidence intervals, effect sizes, degrees of freedom and *P* value noted<br>*Give P values as exact values whenever suitable.*
☒	☐	For Bayesian analysis, information on the choice of priors and Markov chain Monte Carlo settings
☒	☐	For hierarchical and complex designs, identification of the appropriate level for tests and full reporting of outcomes
☒	☐	Estimates of effect sizes (e.g. Cohen's *d*, Pearson's *r*), indicating how they were calculated

Our web collection on statistics for biologists contains articles on many of the points above.

Software and code

Policy information about availability of computer code

Data collection	MSD Sector Imager 6000 was used to develop MSD ELISA plates. Cryo-EM data were collected using SerialEM v3.8.2 and v3.8.18.
Data analysis	Statistical analysis of the data was performed using GraphPad Prism 8. SDS-PAGE gels and western blots were analysed using Image Quant TL v8.1. Cryo-EM image processing and data analysis was performed using MotionCor2 1.4.258, CTFFIND v4.1.14, crYOLO v1.760, RELION3.1, cryoSPARC v3.2.0 and v3.3.1, SIDESPLITTER, UCSF Chimera, Coot 0.9.8, PHENIX 1.19.2, MolProbity, ColabFold and UCSF ChimeraX v1.4. Sequence alignment was performed using Clustal Omega.

For manuscripts utilizing custom algorithms or software that are central to the research but not yet described in published literature, software must be made available to editors and reviewers. We strongly encourage code deposition in a community repository (e.g. GitHub). See the Nature Portfolio guidelines for submitting code & software for further information.

Data

Policy information about availability of data

All manuscripts must include a data availability statement. This statement should provide the following information, where applicable:

- Accession codes, unique identifiers, or web links for publicly available datasets
- A description of any restrictions on data availability
- For clinical datasets or third party data, please ensure that the statement adheres to our policy

The cryo-EM density maps and atomic coordinates generated in this study have been deposited in the EMDB and the PDB under accession codes EMD-17112 (apo

GSEC1B), EMD-17113 (GSEC1B-Aβ46), PDB ID 8OQY (apo GSEC1B) and PDB ID 8OQZ (GSEC1B-Aβ46). Publicly available PDB entries used in this study are available under accession codes 5FN2, 5FN5, 6IDF, 6IYC, 6LQG, 6LR4, 7C9I, 7Y5T.

Research involving human participants, their data, or biological material

Policy information about studies with human participants or human data. See also policy information about sex, gender (identity/presentation), and sexual orientation and race, ethnicity and racism.

Reporting on sex and gender	N/A
Reporting on race, ethnicity, or other socially relevant groupings	N/A
Population characteristics	N/A
Recruitment	N/A
Ethics oversight	N/A

Note that full information on the approval of the study protocol must also be provided in the manuscript.

Field-specific reporting

Please select the one below that is the best fit for your research. If you are not sure, read the appropriate sections before making your selection.

☒ Life sciences ☐ Behavioural & social sciences ☐ Ecological, evolutionary & environmental sciences

For a reference copy of the document with all sections, see nature.com/documents/nr-reporting-summary-flat.pdf

Life sciences study design

All studies must disclose on these points even when the disclosure is negative.

Sample size	The size of the collected cryo-EM dataset was defined by availability of suitable areas for collection of high-contrast cryo-EM images and availability of the microscope time. Sample sizes for biochemical and cell-based assays were chosen to provide significant and reproducible signals using ELISA. No sample size calculation was performed. This was previously described in Petit, Gutiérrez-Fernández et al., 2022.
Data exclusions	During analysis of single particle cryo-EM data a standard exclusion practice was followed. Micrographs with high drift, thick ice and large contaminated areas were excluded. Particles were removed if they were assigned to 2D or 3D classes that resulted in poor reconstructions. For biochemical assays no data were excluded.
Replication	Single particle cryo-EM data contain internal multiplicity and were analyzed using statistical approaches to validate the results. Those included independent processing of separate halves of the datasets. For this reason, the collection of multiple datasets is not needed to validate the 3D reconstructions. Biochemical and cell-based assays were performed independently at least 3 times without any technical replicates. Detailed information is provided in figure legends.
Randomization	For cryo-EM studies, randomization was applied in the form of random particle selection for calculating independent 3D reconstruction for calculating gold-standard FSC curves used for regularization. For biochemical and cell-based assays the variables analysed dictated the experimental set up and data analysis. No grouping or randomisation was used there.
Blinding	Blinding was not used in this study. The results are quantitative and are not a subject of a subjective judgment, and blinding is generally not used in the field.

Reporting for specific materials, systems and methods

We require information from authors about some types of materials, experimental systems and methods used in many studies. Here, indicate whether each material, system or method listed is relevant to your study. If you are not sure if a list item applies to your research, read the appropriate section before selecting a response.

Materials & experimental systems

n/a	Involved in the study
☐	☒ Antibodies
☐	☒ Eukaryotic cell lines
☒	☐ Palaeontology and archaeology
☒	☐ Animals and other organisms
☒	☐ Clinical data
☒	☐ Dual use research of concern
☒	☐ Plants

Methods

n/a	Involved in the study
☒	☐ ChIP-seq
☒	☐ Flow cytometry
☒	☐ MRI-based neuroimaging

Antibodies

Antibodies used

1. anti-NCT (BD Biosciences Cat# 612290, RRID:AB_399607) 1 in 2000
2. anti-PSEN1NTF (Millipore Cat# MAB1563, RRID:AB_11215630) 1 in 1000
3. anti-PSEN1CTF (Cell Signaling Technology Cat# 5643, RRID:AB_10706356) 1 in 1000
4. anti-APH-1B (B78; kindly provided by prof. Bart de Strooper) 1 in 1000
5. anti-PEN-2 (Cell Signaling Technology Cat# 8598, RRID:AB_11127393) 1 in 500
6. goat anti-mouse IgG-HRP conjugate (Bio-Rad Cat# 1721011, RRID:AB_2617113) 1 in 10000
7. rabbit anti-rat IgG-HRP conjugate (Thermo Fisher Scientific Cat# 61-9520, RRID:AB_2533945) 1 in 2000
8. goat anti-rabbit IgG-HRP conjugate (Bio-Rad Cat# 172-1019, RRID:AB_11125143) 1 in 10000
9. anti-NCT (9C3; kindly provided by Prof. Wim Annaert) 1 in 2000
10. anti-human Ab37 (JRD/Ab37/3; Janssen Pharmaceutica NV)
11. anti-human Ab38 (JRF AB038; Janssen Pharmaceutica NV)
12. anti-human Ab40 (JRF/cAb40/28; Janssen Pharmaceutica NV)
13. anti-human Ab42 (JRF/cAb42/26; Janssen Pharmaceutica NV)
14. anti-beta-Amyloid, 1-16 (BioLegend Cat# 803016, RRID:AB_2565329)
15. anti-human Ab43 (27710, IBL)

Validation

Antibodies were validated by the manufacturer, or for the non-commercial ones, validated by the laboratory that generated them. References describing the relevant use of the antibodies are provided below:

1. Chen F, Yu G, Arawaka S, et al. Nicastrin binds to membrane-tethered Notch. Nat Cell Biol. 2001; 3(8):751-754.; Yu G, Nishimura M, Arawaka S, et al. Nicastrin modulates presenilin-mediated notch/glp-1 signal transduction and betaAPP processing. Nature. 2000; 407(6800):48-54.
2. Sebastien Mosser, Jean-René Alattia, Mitko Dimitrov, Alexandre Matz, Justine Pascual, Bernard L. Schneider, Patrick C. Fraering, The adipocyte differentiation protein APMAP is an endogenous suppressor of Aβ production in the brain, Human Molecular Genetics, Volume 24, Issue 2, 15 January 2015, Pages 371–382; Nadav Elad, Bart De Strooper, Sam Lismont, Wim Hagen, Sarah Veugelen, Muriel Arimon, Katrien Horré, Oksana Berezovska, Carsten Sachse, Lucía Chávez-Gutiérrez; The dynamic conformational landscape of γ-secretase. J Cell Sci 1 February 2015; 128 (3): 589–598.
3. Kim, N., Ju, I.G., Jeon, S.H. et al. Inhibition of microfold cells ameliorates early pathological phenotypes by modulating microglial functions in Alzheimer’s disease mouse model. J Neuroinflammation 20, 282 (2023).; Maniv, I., Sarji, M., Bdarneh, A. et al. Altered ubiquitin signaling induces Alzheimer’s disease-like hallmarks in a three-dimensional human neural cell culture model. Nat Commun 14, 5922 (2023).
4. Acx H, Chávez-Gutiérrez L, Serneels L, Lismont S, Benurwar M, Elad N, De Strooper B. Signature amyloid β profiles are produced by different γ-secretase complexes. J Biol Chem. 2014 Feb 14;289(7):4346-55.; Serneels L, Narlawar R, Perez-Benito L, Municoy M, Guallar V, T'Syen D, Dewilde M, Bischoff F, Fraiponts E, Tresadern G, Roevens PWM, Gijsen HJM, De Strooper B. Selective inhibitors of the PSEN1-gamma-secretase complex. J Biol Chem. 2023 Jun;299(6):104794.
5. Choi M, Ryu J, Vu HD, Kim D, Youn YJ, Park MH, Huynh PT, Hwang GB, Youn SW, Jeong YH. Transferrin-Conjugated Melittin-Loaded L-Arginine-Coated Iron Oxide Nanoparticles for Mitigating Beta-Amyloid Pathology of the 5XFAD Mouse Brain. Int J Mol Sci. 2023 Oct 6;24(19):14954.; Jo KW, Lee D, Cha DG, Oh E, Choi YH, Kim S, Park ES, Kim JK, Kim KT. Gossypetin ameliorates 5xFAD spatial learning and memory through enhanced phagocytosis against Aβ. Alzheimers Res Ther. 2022 Oct 21;14(1):158.
6. Ramamoorthi G, Kodumudi K, Snyder C, Grover P, Zhang H, Greene MI, Basu A, Gallen C, Wiener D, Costa RLB, Han HS, Koski G, Czerniecki BJ. Intratumoral delivery of dendritic cells plus anti-HER2 therapy triggers both robust systemic antitumor immunity and complete regression in HER2 mammary carcinoma. J Immunother Cancer. 2022 Jun;10(6):e004841.; Zhang YF, Wang Q, Su YY, Wang JL, Hua BJ, Yang S, Feng JX, Li HY. PPAR?γ agonist rosiglitazone protects rat peritoneal mesothelial cells against peritoneal dialysis solution?induced damage. Mol Med Rep. 2017 Apr;15(4):1786-1792.
7. Nakamura A, Morise J, Yabuno-Nakagawa K, Hashimoto Y, Takematsu H, Oka S. Site-specific HNK-1 epitope on alternatively spliced fibronectin type-III repeats in tenascin-C promotes neurite outgrowth of hippocampal neurons through contactin-1. PLoS One. 2019 Jan 10;14(1):e0210193. ; Chan SM, Sapir T, Park SS, Rual JF, Contreras-Galindo R, Reiner O, Markovitz DM. The HERV-K accessory protein Np9 controls viability and migration of teratocarcinoma cells. PLoS One. 2019 Feb 28;14(2):e0212970.
8. Neumann B, Angstwurm K, Linker RA, Knoll G, Eidenschink L, Rubbenstroth D, Schlottau K, Beer M, Schreiner P, Soutschek E, Böhmer MM, Lampl BMJ, Pregler M, Scheiter A, Evert K, Zoubaa S, Riemenschneider MJ, Asbach B, Gessner A, Niller HH, Schmidt B, Bauswein M. Antibodies against viral nucleo-, phospho-, and X protein contribute to serological diagnosis of fatal Borna disease virus 1 infections. Cell Rep Med. 2022 Jan 18;3(1):100499; Sarikhani M, Garbern JC, Ma S, Sereda R, Conde J, Krähenbühl G, Escalante GO, Ahmed A, Buenrostro JD, Lee RT. Sustained Activation of AMPK Enhances Differentiation of Human iPSC-Derived Cardiomyocytes via Sirtuin Activation. Stem Cell Reports. 2020 Aug 11;15(2):498-514.
9. Esselens C, Oorschot V, Baert V, Raemaekers T, Spittaels K, Serneels L, Zheng H, Saftig P, De Strooper B, Klumperman J, Annaert W. Presenilin 1 mediates the turnover of telencephalin in hippocampal neurons via an autophagic degradative pathway. J Cell Biol. 2004 Sep 27;166(7):1041-54.; Chávez-Gutiérrez L, Tolia A, Maes E, Li T, Wong PC, de Strooper B. Glu(332) in the Nicastrin ectodomain is essential for gamma-secretase complex maturation but not for its activity. J Biol Chem. 2008 Jul 18;283(29):20096-105.
10. -15. Petit D, Hitzenberger M, Koch M, Lismont S, Zoltowska KM, Enzlein T, Hopf C, Zacharias M, Chávez-Gutiérrez L. Enzyme-

substrate interface targeting by imidazole-based γ-secretase modulators activates γ-secretase and stabilizes its interaction with APP. EMBO J. 2022 Nov 2;41(21):e111084.; Koch M, Enzlein T, Chen SY, Petit D, Lismont S, Zacharias M, Hopf C, Chávez-Gutiérrez L. APP substrate ectodomain defines amyloid-β peptide length by restraining γ-secretase processivity and facilitating product release. EMBO J. 2023 Dec 1;42(23):e114372.

Eukaryotic cell lines

Policy information about cell lines and Sex and Gender in Research

Cell line source(s)	Sf9 (B85502; ThermoFisher Scientific)<br>High Five (11496015; ThermoFisher Scientific)<br>PSEN1/PSEN2-deficient MEF cell line was kindly provided by Prof. Bart de Strooper (Herreman et al., 2000)<br>HEK293T (CRL-3216; ATCC)
Authentication	The PSEN1/PSEN2-deficient MEF cell line was validated using Western blot. Other cell lines were validated by sequencing.
Mycoplasma contamination	All cell lines tested negative for mycoplasma contamination.
Commonly misidentified lines<br>(See ICLAC register)	No misidentified cell lines (according to ICLAC Register of Misidentified Cell Lines version 12) were used in this study.
